# Optimizing Endoscopic Transpyloric Feeding Tube Placement in Low Birth Weight Infants: Practical Insights from Clinical Experience

**DOI:** 10.3390/medicina61081481

**Published:** 2025-08-18

**Authors:** Yeoun Joo Lee, Hansol Kim, Shin Yun Byun, Narae Lee, Mun Hui Jung, Seung Hee Jeong

**Affiliations:** Department of Pediatrics, Pusan National University Children’s Hospital, Pusan National University School of Medicine, Yangsan 50612, Republic of Korea

**Keywords:** low birth weight infants, transpyloric tube, enteral nutrition, endoscopic placement, feeding intolerance

## Abstract

*Background and Objectives*: Transpyloric (TP) feeding tube placement is a viable nutritional strategy in low birth weight infants (LBWIs) with severe gastroesophageal reflux or feeding intolerance. However, technical challenges are encountered in patients of this age group due to their small body size and the limited availability of appropriately sized equipment. *Materials and Methods:* We retrospectively reviewed 15 endoscopic TP tube placements performed in 12 LBWIs weighing less than 2.5 kg between May 2017 and March 2025. Emphasis was placed on procedural techniques, equipment selection, and troubleshooting strategies for successful bedside execution. *Results*: All procedures were performed without the use of additional accessories, by advancing a feeding tube preloaded with a guidewire under direct visualization provided by a 5.5 mm outer diameter endoscope. All procedures were technically successful, including 14 performed at the bedside using a portable endoscope. A 6 or 8 Fr feeding tube loaded with a soft-tipped guidewire was advanced through the pylorus under direct endoscopic visualization. The average body weight at the time of the procedure was 1950 ± 296 g. No complications such as mucosal injury, perforation, or tube dislodgement occurred during the procedure. The average enteral feeding volume increased from 33.4 ± 52.8 cc/kg to 92.0 ± 44.4 cc/kg within 7 days. Full enteral nutrition was achieved in all surviving patients within three weeks. The feeding tube remained in place for a mean duration of 26.1 ± 19.2 days. *Conclusions*: Endoscopic TP tube placement in LBWIs can be safely and reliably performed at the bedside with appropriate technical modifications. It facilitates earlier advancement to full enteral nutrition and may serve as a viable option for LBWIs unresponsive to standard feeding methods.

## 1. Introduction

Low birth weight infants (LBWIs), defined as those with birth weight less than 2500 g, are highly susceptible to gastrointestinal complications, including gastroesophageal reflux (GER), vomiting, excessive gastric residuals, and aspiration. These conditions often lead to aspiration pneumonia and pose significant barriers to ventilator weaning in preterm infants, who are already at increased risk of respiratory distress syndrome and bronchopulmonary dysplasia (BPD) [1]. Furthermore, prolonged interruption of enteral feeding increases the risk of total parenteral nutrition (TPN)-associated liver disease and cholestasis and necessitates extended use of central venous catheters, thereby elevating the risk of infection, nutritional deficiencies, and necrotizing enterocolitis [2]. These factors contribute to prolonged hospitalization and poor clinical outcomes [3].

TP feeding tube placement is a well-established therapeutic approach for patients with severe gastroparesis or gastroesophageal reflux (GER) who are unable to tolerate oral or gastric enteral nutrition in both children and adults [4,5,6]. While a Cochrane review published over two decades ago concluded that the potential benefits of TP tube feeding on growth were outweighed by procedure-related risks, suggesting limited utility in preterm infants [7]. However, more recent studies have reported that early TP tube feeding may offer a safer alternative to nasogastric (NG) feeding, particularly in extremely low birth weight infants (ELBWIs), by significantly reducing respiratory-related adverse events [1,8]. These findings suggest that, in the absence of procedural complications, TP tube placement may serve as an effective strategy for achieving successful enteral nutrition in this vulnerable population.

While endoscopic TP tube placement is generally considered safe and effective in pediatric patients, its application in LBWIs remains limited due to technical challenges. The narrow pharynx and esophagus in these LBWIs raise concerns regarding the feasibility and tolerability of endoscopic procedures.

Our institution has accumulated substantial experience in performing endoscopic TP tube placements in neonates and young infants, primarily upon referral from neonatal intensive care unit (NICU) specialists. With a nearly 100% success rate in neonates, the procedure has been extended to include LBWIs weighing over 1400 g from eight years ago, consistently demonstrating excellent clinical performance.

The present study aims to share our institutional experience with endoscopic TP tube placement in LBWIs under 2.5 kg, with particular emphasis on technical considerations, therapeutic efficacy, clinical outcomes, and safety.

## 2. Material and Methods

Between May 2017 and March 2025, we reviewed all cases referred from the NICU to the pediatric gastrointestinal department for endoscopic procedures. Among these, we included only cases in which the patient’s body weight at the time of the procedure was less than 2.5 kg (LBWI) and the indication was endoscopic TP feeding tube insertion. Cases with a body weight ≥ 2.5 kg or those in which endoscopy was performed for diagnostic purposes or for therapeutic interventions such as balloon dilatation for strictures were excluded. Data collected included patient age, weight, indication for TP tube placement, duration of TP tube usage, changes in the amount of enteral nutrition before and after the procedure, complications, and clinical outcomes. Electrical medical records were reviewed, retrospectively. Full enteral nutrition was defined as achieving an enteral feeding volume of more than 100 cc/kg/day without the need for parenteral nutritional supplementation due to vomiting, reflux, or gastric residuals.

Changes in enteral feeding volumes before and after the procedure were analyzed using both paired *t*-tests and Wilcoxon signed-rank tests, with the day (D) of the procedure serving as the reference point. Comparisons were made between 14 days (D − 14) and 7 days (D − 7) prior to the transpyloric tube insertion, and 7 days (D + 7) and 14 days (D + 14) after the procedure. A *p*-value of <0.05 was considered statistically significant. Statistical analyses were performed using SPSS Statistics, version 29; IBM Corp., Armonk, NY, USA.

**Figure 1:**
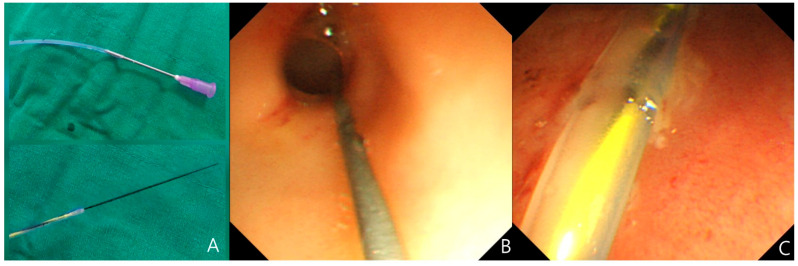
Endoscopic technique for transpyloric (TP) tube placement in low birth weight infants. (**A**) A photograph showing the insertion of a soft-tipped guidewire through the distal end hole of the feeding tube, achieved by puncturing the tube tip with a needle. (**B**) A photograph demonstrating the guidewire successfully advanced through the pylorus. (**C**) A photograph demonstrating the advancement of the feeding tube over the guidewire.

The detailed methods of endoscopic TP tube placement and management protocols for LBWIs were as follows:

Prior to the procedure, a 6 or 8 French feeding tube (Bicakcilar Levin feeding catheter, Bicakcilar Medical Devices, Istanbul, Turkey), a soft-tipped guidewire for ERCP (VisiGlide2^®^, 0.025 inches, straight type; Olympus, Tokyo, Japan), endoscopic equipment (Olympus GIF-XP260N, Olympus Corporation, Tokyo, Japan), and sedation medications (midazolam and ketamine) were prepared. An end hole was created at the distal tip of the feeding tube using a needle, if not pre-existing. Sufficient lubricant was applied to the inside of the tube, and the guidewire was advanced through the lumen of the tube (Figure 1A). For the procedure, after adequate sedation (0.1 mg/kg midazolam and/or 1 mg/kg ketamine), the feeding tube was first inserted through the patient’s mouth or nose. The endoscope was then introduced orally after applying adequate lubrication. With the pylorus clearly visualized from a frontal view, the guidewire was inserted carefully beyond the pylorus (Figure 1B). The feeding tube was subsequently advanced over the guidewire and passed through the pylorus into the small intestine (Figure 1C). Following successful placement, the endoscope was gently withdrawn, and the guidewire was subsequently removed. A plain abdominal radiograph was obtained to verify the position and depth of the feeding tube. If the feeding tube was excessively coiled in the stomach, adjustment could be made by gently withdrawing the tube.

Thereafter, imaging was performed the following day at the discretion of the attending clinician, and subsequently only when tube displacement or dysfunction was suspected. When a chest X-ray was clinically indicated, infantography was performed instead of the chest X-ray to confirm the tube position in the abdomen. While the TP tube remained in the duodenum or jejunum, it was flushed with clear water every 4 h to prevent clogging. If medication administration was required, it was delivered through a separate nasogastric feeding tube.

*Ethics Statement*

This study was approved by the Institutional Review Board of Pusan National University Yangsan Hospital (IRB No.55-2025-039, date: 10 April 2025), and the requirement for informed consent was waived due to its retrospective nature.

## 3. Results

A total of 12 patients (male:female = 7:5) underwent 15 transpyloric (TP) feeding tube placement procedures. The mean gestational age at birth was 30 ± 2 weeks, and all patients were born prematurely. The mean age at the time of the procedure was 42.3 ± 20.8 days (range: 16–78). The mean birth weight was 1158 ± 483 g (range: 590–2110), and the mean body weight at the time of endoscopy was 1950 ± 296 g (range: 1410–2460). Notably, five patients weighed less than 2000 g at the time of the procedure.

All 12 patients had failed to achieve full enteral nutrition prior to the procedure due to persistent vomiting, severe regurgitation, or large gastric residuals, which were unresponsive to conventional treatments for underlying conditions such as gastroesophageal reflux (GER) or gastroparesis. Two patients were in a postoperative state: one had developed a hiatal hernia following surgery for congenital diaphragmatic hernia, and the other had undergone surgery for jejunal atresia.

Of the 15 procedures, 14 were performed at the bedside in the NICU using portable endoscopic equipment, while one was conducted in the endoscopy suite (performed in a 78-day-old, 2460 g patient in the postoperative state after jejunal atresia repair). Seven patients were already intubated due to their underlying medical conditions; none required elective intubation for the purpose of TP tube placement.

All 15 endoscopic TP tube placements were technically successful, and no procedure-related complications were observed. Post-procedurally, the tip of the TP tube was positioned in the duodenum in six cases and in the proximal jejunum in nine cases. The average duration of TP tube therapy was 26.1 ± 19.2 days (range: 3–70).

Two patients required repeated procedures. In one case, early removal due to vomiting necessitated re-placement on day 3. In the other case, the patient maintained the TP tube in the jejunum for a total of 70 days, with additional endoscopic re-placements on day 36 and day 21 due to tube leakage. Unintentional removal occurred in one duodenal case due to vomiting and in one jejunal case during extubation. The average duration of TP tube therapy was 20.0 ± 12.2 days for duodenal tubes and 23.3 ± 14.3 days for jejunal tubes.

**Figure 2:**
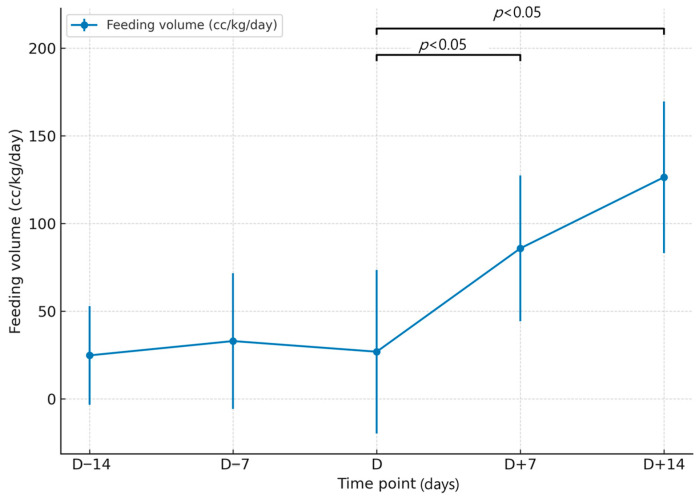
Changes in mean enteral feeding volume (cc/kg/day) over time relative to the day of the transpyloric feeding tube insertion (D). Error bars indicate standard deviations. *p* < 0.05 compared with D (paired *t*-test and Wilcoxon signed-rank test).

Regarding nutritional outcomes, the average enteral feeding volume prior to TP tube placement was 33.4 ± 52.8 cc/kg (range: 0–153.0). Seven days after the procedure, the volume increased to 92.0 ± 44.4 cc/kg (range: 31.9–161.5) in 11 patients. At two weeks post-placement, 9 out of 11 patients had reached over 100 cc/kg of enteral feeding. Full enteral nutrition was achieved in all surviving patients within three weeks of the procedure (Table 1, Figure 2).

When comparing pre- and post-procedure volume of enteral nutrition relative to the day of the TP tube insertion day, neither D − 14 vs. D nor D − 7 vs. D showed statistically significant differences based on the paired *t*-test (*p* = 0.810 and *p* = 0.532, respectively) or Wilcoxon signed-rank test (*p* = 0.678 and *p* = 0.922, respectively). In contrast, both D vs. D + 7 and D vs. D + 14 demonstrated significant increases in enteral feeding volumes. The paired *t*-test showed *p* < 0.001 for both comparisons (*t* = −6.654 and *t* = −5.985), and the Wilcoxon signed-rank test also confirmed significance (*p* = 0.002 and *p* = 0.0077, respectively) (Table 2).

One patient died three days after the procedure due to underlying respiratory failure unrelated to the intervention. The remaining 11 patients achieved full enteral nutrition and survived.

## 4. Discussion

There are several methods for transpyloric feeding tube (TP tube) placement [9], including endoscopic placement [10], fluoroscopic guidance [10,11], placement using a magnet-tipped tube [12], blind placement assisted by prokinetic agents [13], ultrasound-guided placement [14], and surgical options. Each method demonstrates varying success rates. In young pediatric patients, fluoroscopic-guided placement is commonly used with a high success rate [11]; however, it requires transporting the patient to the fluoroscopy suite, along with radiation exposure. Additionally, this method is also difficult for premature or low birth weight infants who have difficulty regulating their body temperature, or patients who are receiving mechanical ventilation. These patients have difficulty moving, making this procedure difficult and less feasible. Electromagnetic-guided placement requires specially designed tubes, limiting its general use, especially in children. Blind placement has a relatively variable success rate of 70% to 100% in newborns and infants [15] and often necessitates repeated X-ray confirmation, making it less ideal. Ultrasound-guided placement [14] is considered an ideal radiation-free, bedside method when performed by a highly skilled operator. However, to ensure adequate visualization, the stomach must be filled with fluid, which may increase the risk of regurgitation in patients with impaired gastric emptying.

Endoscopic placement [10] offers a high success rate and, with the portability of endoscopic equipment, can be performed at the bedside. Additionally, it allows for the detection of other gastrointestinal problems during the procedure, such as antral webs, pyloric strictures, or inflammatory conditions of the stomach and duodenum.

In general, endoscopic placement of a transpyloric feeding tube involves using forceps to grasp the tube directly within the stomach or pulling a suture tied to the tube to guide it past the pylorus.

There are several endoscopic methods for transpyloric (TP) tube placement, and no single technique has been proven to be superior. Commonly used approaches involve directly grasping the tube within the stomach using a large-caliber forceps or snare and advancing it through the pylorus. However, in very small infants, the gastric angle is often extremely abrupt, requiring a degree of endoscopic angulation comparable to a J-turn in order to visualize the pylorus. Furthermore, the use of forceps is often impractical due to the narrow diameter of the pediatric endoscope, making this technique less feasible in smaller children.

Chang et al. reported a technique in which a suture tie is created at the distal tip of the feeding tube, allowing it to be grasped with biopsy forceps and advanced into the duodenum. This method demonstrated a success rate of 97% [16]. We also employ this technique when inserting an 8 Fr tip-weighted feeding tube in children weighing over approximately 5 kg and have found it to be highly useful. However, in patients with a narrow duodenum, the use of forceps carries a higher risk of mucosal injury, making this method less suitable for very small infants. Wiggin and Delegge et al. reported the insertion of a 12-French nasoenteric tube (NET) preloaded with a Savary wire as a nasojejunal tube under endoscopic visualization. They achieved a 100% success rate with a mean procedure time of 11.1 ± 2.2 min, demonstrating both a very rapid insertion time and a high success rate [17]. The Savary wire features a flexible tip but a relatively stiff shaft, which allows for effective transmission of force. This characteristic helps prevent the tube from coiling within the stomach during insertion. However, the Savary wire is too rigid for use in small children and cannot be inserted into feeding tubes with a narrow internal diameter.

Therefore, the authors devised a technique using a biliary catheter, which is softer than a Savary wire yet retains sufficient stiffness, to advance a conventional nasogastric or orogastric feeding tube. This method offers the advantage of utilizing standard feeding tubes and can be applied as a simple technique without the need for additional instruments such as forceps or snares. Before attempting the procedure in LBWIs, the authors gained experience by performing multiple successful TP tube placements in smaller infants weighing around 3 kg. In these cases, the tube was inserted using the same technique as described in this study—without the use of forceps. This approach yielded a high success rate and allowed the authors to perform the procedure in LBWIs without experiencing technical failure.

The first key point in this technique is to use a soft guidewire that passes through the “end” hole of the feeding tube. If the guidewire passes through a side hole instead of the end hole, even after the wire may pass through the pylorus, the feeding tube tip may have difficulty advancing past the pylorus. The second key point is to prevent the feeding tube from being dislodged when retrieving the endoscope. In pediatric patients with a narrower gastrointestinal lumen, the endoscope and the tube can frequently come out together during withdrawal after successful transpyloric tube placement. To avoid this, it is crucial to “adequately lubricate” the outer surface of the feeding tube. Additionally, firmly holding the guidewire-loaded tube in place while withdrawing the endoscope with a slight “snap” motion can be helpful. Lastly, when using the feeding tube, it is important to adequately flush the tube every 4 h. Feeding tubes have a very small diameter, which increases the risk of tube occlusion. Additionally, it is recommended that medications be administered separately via a dedicated nasogastric tube, rather than through the transpyloric feeding tube. In contrast, since low birth weight infants (LBWIs) exhibit relatively limited movement, the risk of tube migration is comparatively low without external manipulation in our experience.

We have previously shared this method with colleagues in small professional gatherings, such as expert case conferences, providing instructional materials and videos, and have subsequently observed many colleagues adopting it in their own hospitals. This approach is not intended to showcase exceptional endoscopic skill, but rather to introduce a practical methodology for feeding tube placement with a preloaded guidewire under direct endoscopic visualization. Unlike the conventional over-the-wire technique, in which there is no reliable way to confirm guidewire position without fluoroscopy, our method allows tube advancement without removing the endoscope, enabling continuous visualization to ensure accurate placement. With the appropriate equipment, it should be readily performed by pediatric gastroenterologists who are already proficient in diagnostic upper gastrointestinal endoscopy in children and neonates, at least in those with normal anatomical structures.

A previous retrospective study measured esophageal diameter in children using contrast-enhanced radiographic imaging to establish age- and weight-based reference values [18]. Based on the linear regression model from that study, esophageal diameter can be estimated as a function of body weight in kilograms. For example, an infant weighing approximately 2 kg is expected to have an esophageal diameter in the range of 5–8 mm. The outer diameter of the smallest gastroscope currently available at our institution is 5.5 mm. Based on this reference and technical feasibility, we initiated endoscopic transpyloric feeding tube placement in low birth weight infants, assuming the procedure could be safely performed in this population.

Despite being a retrospective review from a single institution and involving a limited number of patients treated with a single technique, the present study aims to highlight the successful experience of a practice implemented in real-world clinical settings.

## 5. Conclusions

Transpyloric (TP) feeding tube placement facilitates earlier achievement of full enteral nutrition in low birth weight infants (LBWIs) and is also useful in identifying concomitant gastrointestinal abnormalities. Although it is an invasive procedure, endoscopic TP tube placement can be safely performed in LBWIs—even in those weighing as little as 1500 g—when conducted by an experienced pediatric gastroenterologist. This technique may be considered a feasible and effective option for nutritional support in LBWIs with severe gastroparesis or gastroesophageal reflux disease (GERD) unresponsive to conventional treatments.

## Figures and Tables

**Table 1:** Patient demographics and feeding amounts before and after TP tube insertion.

	At the Time of TP Tube Insertion	1 Week After TP Tube Insertion	2 Weeks After TP Tube Insertion
Rate of over 100 cc/kg of enteral nutrition	3/12 patients	3/11 ^†^ patients	9 */11 ^†^ patients
Average volume of enteral nutrition (cc/kg, mean ± SD)	33.4 ± 52.8 (range: 0.0–153.0)	92.0 ± 44.4 (range: 31.9–161.5)	129.5 ± 42.3 (range: 33.3–183.3)
Average body weight (grams, mean ± SD)	1965 ± 303	2173 ± 334	2307 ± 383

* Two patients reached full enteral nutrition 3 weeks after TP tube insertion. ^†^ One patient died due to underlying disease (severe bronchopulmonary dysplasia).

**Table 2:** Comparison of average volume of enteral nutrition before and after TP tube insertion.

Comparison (*n* = 10 *)	Paired *t*-Test *t*-Value	Paired *t*-Test *p*-Value	Wilcoxon W-Value	Wilcoxon *p*-Value
D − 4 vs. D	−0.247	0.810	19.0	0.678
D − 7 vs. D	0.649	0.532	26.0	0.922
D vs. D + 7	−6.654	<0.001	0	0.002
D vs. D + 14	−5.985	<0.001	0	0.0077

* Of the 12 patients, one was excluded because the procedure was performed immediately after transfer and data for D − 14 and D − 7 were unavailable; another was excluded due to death after the procedure.

## Data Availability

The data presented in this study are available on request from the corresponding author. The data are not publicly available due to institutional policy.
